# Potential susceptibility genes in patients with stage III and IV periodontitis: A whole-exome sequencing pilot study

**DOI:** 10.17305/bb.2023.9282

**Published:** 2024-02-01

**Authors:** Fan Xue, Jianjiang Zhao, XiaoCui Gao, Xuehai Jiang, Zedong Lan

**Affiliations:** 1 Department of Periodontics, Shenzhen Stomatological Hospital, Southern Medical University, Shenzhen, China

**Keywords:** Periodontitis, whole-exome sequencing (WES), genetic susceptibility, single nucleotide polymorphism (SNP), insertion/deletion (InDel), single nucleotide variant (SNV)

## Abstract

The aim of this study was to screen potential susceptibility genes using whole-exome sequencing (WES) in 15 Han Chinese patients with stage III or IV periodontitis and to evaluate the quantity and quality of genomic DNA extracted from saliva. DNA was extracted from saliva epithelial cells, quality-tested, and then subjected to WES and bioinformatics analyses. All variation loci were analyzed and interpreted following the American College of Medical Genetics and Genomics (ACMG) criteria. Candidate pathogenic variation loci were identified and verified using Sanger sequencing. Correlation and functional analyses of the candidate genes were used to identify potential susceptibility genes in patients with severe periodontitis. *LFNG*, *LENG8*, *NPHS1, HFE*, *ILDR1*, and *DMXL2* genes were identified in over two cases each with shared mutations. Following these analyses, the *DMXL2* gene was identified as being associated with stage III and IV periodontitis. These results suggest a potential pathophysiological risk mechanism for periodontitis, but need to be verified through larger clinical studies and mechanistic experiments to determine the pathogenicity of these gene mutations and their generalizability to a wider population of periodontitis patients. By screening candidate pathogenic variation loci using WES in 15 Han Chinese patients with stage III or IV periodontitis, our study could provide a pipeline and feasibility support for the identification of susceptibility genes in patients with stage III and IV periodontitis.

## Introduction

Stage III and IV periodontitis is a periodontal disease causing severe chronic inflammation that damages periodontal tissues. However, the severity of damage is not proportional to the local irritants such as plaque. The pathogenesis of severe periodontitis remains unclear and is considered to be related to abnormal host immune-inflammatory responses. Severe periodontitis has a prevalence rate of 11% globally [1], and is considered a polygenic disease with an estimated heritability of 50% [1]. There is growing research evidence that the development of severe periodontitis is associated with the presence of susceptibility genes and that individual genetic susceptibility factors play an important role in its onset and progression. The identification of susceptibility genes will help elucidate the pathogenesis of severe periodontitis.

Currently, the genetic factor of polygenic diseases mostly arise from gene polymorphisms. A gene polymorphism is defined as the presence of two or more genotypes at one gene locus on a chromosome within a randomly mating population at a particular genome position, which results from gene mutations. Sometimes a single nucleotide variant (SNV) is referred to as single nucleotide polymorphism (SNP) if it is present in at least 1% of the population. An SNV can be rare in one population but common in another. SNPs and insertion/deletion polymorphisms (InDel) are the most common polymorphisms. SNPs account for about 90% of DNA sequence variants in the human genome. However, InDels, sequence variations involving the insertion or deletion of sequence that is usually <50 bp, rank second only to SNPs as the most prevalent form of genetic variation. The polymorphism of a certain gene may be associated with specific disease phenotypes, and genetic susceptibility to disease may be enhanced by environmental factors. Alterations of the genetic code due to SNP, SNV, or InDel may lead to changes in the function or release of encoding molecules, which is an important basis for determining human susceptibility to disease. Structural variations are the major source of genomic variation and are markedly correlated with clinical disease phenotypes. Copy number variations (CNVs) are the main type of structural variations, defined as an increase or decrease in the copy number of large-segment sequences (usually >1 kbp) in the genome. Therefore, accurately identifying the polymorphism, locus variation, and the mechanistic roles of related susceptibility genes at the molecular level is critical for understanding the pathogenesis of severe periodontitis and its potential therapeutic targets.

In recent years, the associations between gene polymorphisms and the onset, course, and prognosis of periodontitis have attracted much research attention. Some studies have shown that polymorphisms of *FcγR* [2], *IL1β* [3]*, VDR* [4]*, IL1RN* [5], and *TNFα* [6] genes may be associated with the development of juvenile periodontitis. Polymorphisms of *TLR4* [7–9], *IL6* [7, 10], *IL1β* [3], *MMP1* [11], *IL10* [12, 13], *VDR* [7], *CD14* [14], *HLA* [15, 16], *IL1RN* [5, 17], and *ER* [18] genes, including *FcγR*, are related to the occurrence and infiltration of inflammation. These include important inflammatory mediator genes, such as *TNFα*, *IL6*, *IL10*, and *IL1β*. Moreover, hormone receptor genes *VDR* [4, 7] and *ER*, which regulate alveolar bone metabolism, are associated with susceptibility to chronic periodontitis. Most of the above-mentioned genes associated with susceptibility to periodontitis are inflammation-associated genes and their mediators, which are involved in gingival inflammation and probably dependent on the active inflammation occurring in gingival epithelial cells throughout the course of periodontal disease. The findings of the study also suggested that transglutaminase gene expression may be modified in response to chronic injury in the damaged gingiva and could be associated with the increased expression of *MMP*s [19]. Further, studies showed that melatonin could be a potential endogenous molecule that exerts anti-inflammatory and immunomodulatory actions for periodontitis by reducing the pro-inflammatory proteins (inteleukin 1 beta [IL-1β], IL-6, and tumor necrosis factor alpha [TNF-α]) and improving key periodontal parameters [20, 21]. However, the lack of rich genetic information and studies that have investigated locus variations in severe periodontitis, continues to limit our understanding of the role of genes in periodontitis.

High-throughput sequencing technologies, also known as next-generation sequencing (NGS), can quickly and accurately generate high-throughput data on mutation loci, and help identify new mutation loci, providing an efficient approach to uncovering candidate genes involved in periodontitis. It is now widely recognized that complex polygenic diseases are caused by low-frequency or rare mutations, and that these mutations show large genetic effects that are often not closely linked to conventional SNPs. This may explain the so-called “missing heritability” observed in genome-wide association study (GWAS) arrays, which refers to the discrepancy between the genetic variations identified and their ability to explain heritability. NGS-based whole-exome sequencing (WES) is characterized by the requirement for fewer samples and having higher detection rate for rare alleles, as compared to GWAS, and is primarily used to analyze mutation loci in the genome, including SNVs, SNPs, InDels, and CNVs. WES can be used to find most disease-related mutations in the exon region, including common mutations and low-frequency mutations (<5%). Moreover, WES is suitable not only for the screening of sporadic hereditary polygenic diseases in individuals but also addresses the insensitivity of GWAS to rare mutations and structural mutations, facilitating the discovery of new rare coding variants.

In this pilot study, the aim was to screen potential susceptibility genes using WES in 15 Han Chinese patients with stage III or IV periodontitis. DNA was extracted from saliva, its quality was assessed, and performance was validated by WES and bioinformatics analysis procedures. All variation loci were analyzed and interpreted according to the interpretation rules of American College of Medical Genetics and Genomics (ACMG). The candidate pathogenic variation loci were verified by Sanger sequencing. Furthermore, the secondary objective was to evaluate the quantity and quality of genomic DNA extracted from saliva and to provide a pipeline and feasibility support for a subsequent large-scale population-based case-control study.

## Materials and methods

### Participants

Unrelated patients and control participants who visited the Department of Periodontics in the Shenzhen Stomatological Hospital of Southern Medical University from June to December 2021 were recruited. A total of 35 participants were enrolled in the study, including 15 patients with severe periodontitis and 21 healthy individuals as controls. Based on the clinical and X-ray examinations, all patients included in the present study met the definition of periodontitis stage III or IV with regard to the extent and severity and Grade C due to the early onset of the disease. The criteria included: at least one site with a probing depth (PD) and clinical attachment level (CAL) ≥ 5 mm in incisors and/or first molars and at least 6 teeth with similar PD and CAL measurements, alveolar bone resorption reaching 1/2–2/3 of root length shown by X-ray imaging, number of missing teeth due to periodontitis ≥3, and percentage of alveolar bone resorption at the tooth site with the most rapid progression of periodontal disease as measured by the imaging/age ratio >1. The inclusion criteria were as follows: 1) Han Chinese ethnicity (determined by their ID card or household registration) within three generations, with no kinship confirmed by lineage tracing; 2) age of 15–55 years; 3) patients with severe periodontitis met the diagnostic criteria for stage III or IV and Grade C generalized periodontitis. The following exclusion criteria were established for all participants: 1) local irritants, such as an inappropriate prosthesis and malocclusion; 2) periodontal treatment or related oral treatment in the last three months; 3) antibiotics, immunosuppressants, anti-inflammatory or any other drugs in the last six months before the study; 4) diabetes or rheumatic diseases; 5) lactation or pregnancy; and 6) smoking.

### DNA extraction and whole-exome sequencing (WES)

The oral mucosal epithelial cells are characterized by vigorous metabolism, rapid renewal, and proneness to shedding, and are naturally shed into saliva. Participants were asked to spit 1–2 mL of saliva, containing oral mucosal epithelial cells, into a tube from a saliva DNA collection kit (Zeesan Biotech). The saliva was mixed with a preservation solution and stored at room temperature. DNA was then extracted from the sample. We used gel electrophoresis to determine DNA concentration and quality. DNA quality and concentration were further quantified using a Qubit fluorometer (Thermo Scientific™). WES was performed (Berry Genomics, Beijing) using three exome capture-based methods in flexible combination: Agilent-V6/V7, IDT-39M, and Twist-33M/36.6M. The genomic DNA samples were randomly fragmented into 180–250 bp using enzymes. DNA fragments were end-repaired, A-tailed, and ligated to adapters at both ends for the preparation of DNA libraries. Adapter-ligated libraries were enriched by polymerase chain reaction (PCR) amplification. Exon regions were captured on magnetic beads through hybridization in the liquid-phase system with biotin-labeled capture probes. Exome libraries were enriched in a PCR reaction followed by library quality assessment. Only qualified libraries were sequenced on an Illumina NovaSeq platform for paired-end 150 bp reads.

### WES data analysis

**Figure 1.**
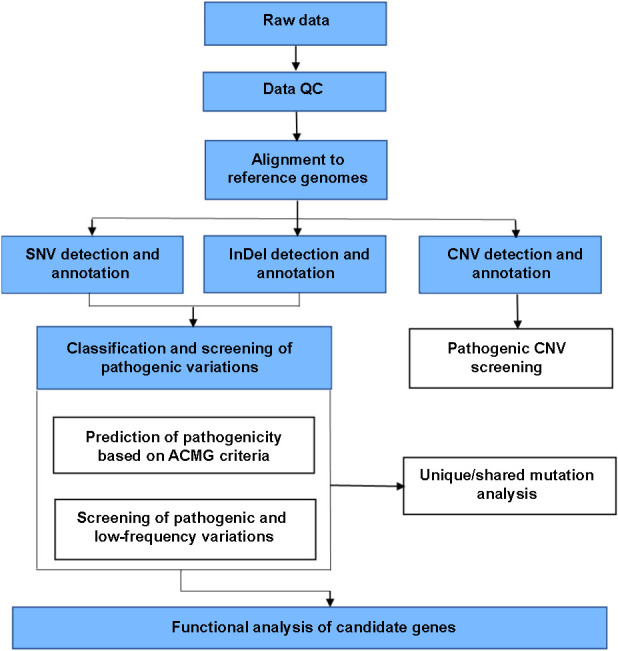
**Flowchart showing the data processing steps in identifying candidate genes.** Classification and screening of variation harmfulness are shown in white boxes. SNV: Single nucleotide variant; CNV: Copy number variation; ACMG: American College of Medical Genetics and Genomics; QC: Quality control.

The original sequencing data (reads) were subjected to quality control checks and processing to remove low-quality reads and then compared with the reference genome (hg19; GRCh37) using BWA software. The initial comparison results in the SAM format were converted to the BAM format using SAMtools software, and then ordered. The repetitive sequences were marked using Picard software, and the basic data and comparison results were recorded. Based on the comparison results, SNVs/SNPs and InDels were detected using GATK software and annotated using the Enliven annotation system independently developed by Berry Genomics, to determine the variation locus, functional regions, and gene information, and to predict the synonymous/nonsynonymous mutations and the negative effects of the amino acid substitutions. Then, the candidate susceptibility genes/mutation loci for severe periodontitis were screened from the final mutation results by the following protocol: 1) mutation loci with high-frequency mutations in the normal population were filtered out using the latest 1000 Genomes Project database (1000g2015aug_all and 1000g2015aug_eas) (https://www.internationalgenome.org/data), the Exome Aggregation Consortium (ExAC) database (ExAC_ALL and ExAC_EAS) (https://exac.broadinstitute.org/), and the Berry Genomics “China Data Cloud” database, which includes data from 400,000 individuals in the Chinese population; loci with a minor allele frequency (MAF) ≤0.05 were retained; 2) synonymous mutations, which theoretically do not alter amino acids (i.e., unlikely to alter protein function), were filtered out, whereas the loci with functional changes were retained; 3) mutation loci without annotated genes were filtered out; 4) the loci of non-synonymous mutations in functional regions, such as Exonic and Splicing (i.e., loci with functional changes) were retained; 5) mutation loci predicted to be harmful by at least two of the four predictive analytic tools (SIFT, PolyPhen, Mutation Taster, and CADD) were retained (this step was not used for InDel screening) (Figure 1).

### Sanger sequencing

Candidate variants identified by WES were verified using Sanger sequencing. Sanger sequencing verification was performed for the susceptibility gene loci in the 15 periodontitis samples and 21 controls, including the candidate variants exon2: c.163_166dupGATG (rs34637446) in *LFNG*, exon2: c. 1-59_1-55delinsGTAGA (rs10407651+TAG+rs1035451) in *LENG8*, exon14: c.1802G>C (rs114615449) and exon11: c.1339G>A (rs28939695) in *NPHS1*, exon2: c.187C>G (rs1799945) in *HFE*, exon6: c.772C>T (rs142746163) in *ILDR1*, and c.88-4_88-3dup (rs3078066) in *DMXL2*. Forward and reverse primers for the exon of the candidate pathogenic variation loci were designed and synthesized by Ige Biotechnology (Guangzhou, China) and used in PCR reactions followed by Sanger sequencing. PCR reactions included 2 µL of extracted genomic DNA with 5U Platinum TaqDNA Polymerase (Applied Biosystems, USA) and 10× buffer supplied by the manufacturer. The PCR primer sequences are shown in Table 1. Amplification conditions were as follows: pre-denaturation at 94 ^∘^C for 5 min, (denaturation at 94 ^∘^C for 30 s, annealing at 56 ^∘^C for 30 s, extension at 72 ^∘^C for 30 s) × 35 cycles, and extension at 72 ^∘^C for 3 min (5 min for the *LFNG* gene). PCR amplification was then performed on DNA samples using the above PCR primers and conditions. The products were visualized on agarose gels and then subjected to Sanger sequencing using an ABI sequencer (ABI, USA). Finally, the sequencing chromatogram was viewed using Chromas Lite software, and the sequencing results were compared using SeqMan software (DNAstar, USA).

**Table 1:** Prediction results of pathogenicity based on ACMG criteria

**Sample No.**	**1**	**2**	**3**	**4**	**5**	**6**	**7**	**8**	**9**	**10**	**11**	**12**	**13**	**14**	**15**
Total	82048	81835	80785	81071	81417	82183	80506	80696	82294	80725	78111	80304	82294	80541	80108
Pathogenic	7	3	4	1	3	5	1	3	5	6	3	3	2	8	7
Likely pathogenic	11	13	13	17	10	18	10	12	8	13	12	16	17	10	16
VUS	6491	6752	6329	6182	6545	6623	6299	6229	6595	6321	5209	6262	6502	6227	5858
VUS-FP	1063	1243	900	950	1156	1272	890	880	1135	904	764	892	1085	900	946
Likely benign	33	54	35	39	40	34	43	45	37	42	41	32	34	43	39
Benign	74443	73770	73504	73882	73663	74231	73263	73527	74514	73439	72082	73099	74654	73353	73242

VUS: Variants of uncertain significance; VUS-FP: Variants of uncertain significance-false positive; ACMG: American College of Medical Genetics and Genomics.

**Figure 2.**
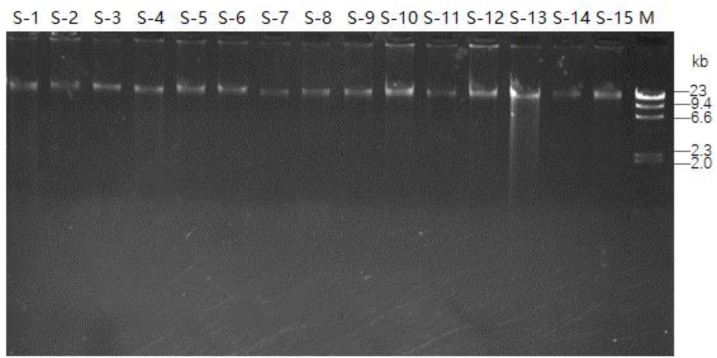
**DNA agarose electrophoresis showing DNA extraction for 15 samples.** The bands on the extreme right are the λ-HindIII DNA marker (Takara Code: D3403A; 125 bp–23,130 bp). The bands from S-1 to S-15 refer to samples 1–15.

### Ethical statement

This study was approved by the human subjects Ethics Board of Shenzhen Stomatological Hospital affiliated to Southern Medical University (Ethics approval number: 2021-02). All data and sample collection procedures in this study were performed in accordance with the Helsinki Declaration of 1975, as revised in 2013. Written informed consent was obtained from all participants.

### Statistical analysis

Two-sided chi-square tests (or adjusted chi-square tests) were used to compare allele frequencies between patients with severe periodontitis and both the control and dbSNP control groups. When the data did not meet the criteria for chi-square tests (or adjusted chi-square test), Fisher’s exact test was used to test for differences between groups. *P* < 0.05 was considered to indicate statistical significance.

## Results

### Clinical assessment of participants

We enrolled 15 patients (cases) with severe periodontitis and 21 healthy controls for this study. All cases and controls were unrelated and of Han Chinese ethnicity from Shenzhen city of Guangdong Province in China. Participants comprised cases (mean age 30.5 ± 9.8 years; 5 males and 10 females) and controls (mean age 28.4 ± 7.6 years; 13 males and 8 females). We found no statistical difference in age (*P* ═ 0.37) or sex (*P* ═ 0.18) between the two groups.

### DNA extraction and quality

Total DNA extracted from saliva samples reached the standard of WES (concentration ≥3 ng/µL, total amount ≥200 ng). The extracted DNA was free of contamination (sample grade 1). The results of DNA agarose electrophoresis are shown in Figure 2.

### WES data analysis

#### WES data

The raw sequencing data output was 305.6 G. The average sequencing depth on target was 115.22× and the coverage of the target region was 97.13% by ≥1× sequencing depth. Fraction of target covered at least 4×, 10×, 20×, and 50×; sequencing depths were 96.78%, 96.17%, 94.89%, and 84.52%, respectively. The data meet the requirements for the intended use.

#### Prediction of pathogenicity based on ACMG criteria

In 2015, the ACMG Genetic Variation Classification Criteria and Guidelines were issued, listing the evidence for pathogenic and benign mutation loci into 28 specific criteria. First, the evidence was classified by type (population data, computational prediction data, functional data, etc.) and strength (supportive, moderate, strong, very strong, or independent). Then, the pathogenicity of mutations was assessed by the “standard combination or maximum combination” of the above-mentioned criteria. According to the ACMG criteria, mutations were further classified based on their harmfulness into pathogenic (P), likely pathogenic (LP), variants of uncertain significance (VUS), false positive (VUS-FP), likely benign, or benign. Using these identified mutations, we focused on those in the P and LP categories as candidate mutation loci in our subsequent analyses (Table 1).

#### Screening of low-frequency and harmful mutations

To more accurately locate the target pathogenic mutations, low-frequency and harmfulness screening was performed on all mutation loci, followed by ACMG grading. The procedures involved in screening for low-frequency and harmful mutations are shown in the study protocol. Following the screening, SNVs and InDels were recorded (Table 2).

**Table 2:** Screening results of low-frequency and harmful mutations based on ACMG criteria

**Sample No.**	**1**	**2**	**3**	**4**	**5**	**6**	**7**	**8**	**9**	**10**	**11**	**12**	**13**	**14**	**15**
Total	1299	1387	1199	1149	1269	1375	1189	1092	1252	1219	1071	1082	1275	1116	1091
Pathogenic	6	3	3	1	3	4	1	3	3	6	2	3	2	7	7
Likely pathogenic	9	11	9	15	10	15	10	12	7	11	10	15	15	8	14
VUS	975	984	911	863	920	965	918	849	925	929	822	834	961	846	782
VUS-FP	130	186	102	113	152	159	91	75	136	91	81	70	130	115	114
Likely benign	3	11	2	3	5	0	3	6	2	5	2	5	3	5	7
Benign	176	192	172	154	179	232	166	147	179	177	154	155	164	135	167

VUS: Variants of uncertain significance; VUS-FP: Variants of uncertain significance-false positive; ACMG: American College of Medical Genetics and Genomics.

#### Copy number variations screening

Using the ACMG classifications, benign CNVs were filtered out from the CNV detection results, and possible pathogenic CNVs were retained. Then, CNV grading was performed by using Berry Genomics according to the ACMG criteria, followed by statistical analysis (Table 3).

**Table 3:** Copy number variation screening results based on ACMG criteria

**Sample No.**	**1**	**2**	**3**	**4**	**5**	**6**	**7**	**8**	**9**	**10**	**11**	**12**	**13**	**14**	**15**
Total	9	4	13	6	10	7	6	10	9	4	7	12	14	7	14
Pathogenic	0	0	0	0	0	0	0	0	0	0	0	0	0	0	0
Likely pathogenic	0	0	0	0	0	0	0	0	0	0	0	0	0	0	0
VUS	4	2	6	1	6	2	3	2	5	0	3	7	5	4	7
Benign	5	2	7	5	4	5	3	8	4	4	4	5	9	3	7

VUS: Variants of uncertain significance; ACMG American College of Medical Genetics and Genomics.

#### Screening results of susceptibility genes

According to the screening for low-frequency and harmful mutations and the pathogenicity prediction results based on the ACMG criteria, we identified over two cases of shared mutations, each in six genes. These included 4 cases with a mutation in *LFNG* (frameshift insertion at exon 2: c.163_166dup), 3 cases in *NPHS1* (nonsynonymous mutations at exon 14: c.1802G>C and exon11: c.1339G>A), 2 cases in *HFE* (nonsynonymous mutations at exon2: c.187C>G in 2 cases), 2 cases in *ILDR1* (stop-gain at exon6: c.772C>T), 2 cases in *LENG8* (splicing at exon2: c.1-59_1-55delinsGTAGA), and 2 cases in *DMXL2* (splicing at c.88-4_88-3dup) (Table 4).

**Table 4:** Screening results of candidate susceptibility genes in 15 patients with severe periodontitis

**Gene**	**Sample count**	**Position**	**Genotype**	**Type**	**Exon**	**hgvs.c**	**ACMG level**
*LFNG*	4	chr7:2552885- 2552885	het	Frameshift insertion	exon2	c.163 166dup	Pathogenic
*NPHS1*	3 3	chr19:3633639 8-36336398 chr19:3633904 4-36339044	het	Nonsynonymous	exon14 exon11	c.1802G>C c.1339G>A	Pathogenic
*HFE*	2	chr6:26091179 -26091179	het	Nonsynonymous	exon2	c.187C>G	Likely pathogenic
*LENG8*	2	chr19:5496246 3-54962467	het	Splicing	exon2	c.1-59 1-55delins	Pathogenic
*ILDR1*	2	chr3:12171303 5-121713035	het	Stop-gain	exon6	c.772C>T	Pathogenic
*DMXL2*	2	chr15:5186838 0-51868380	het	Splicing	–	c.88-4 88-3dup	Pathogenic

Gene: Name of the gene at the mutation locus; Sample count: Number of samples; Type: Mutation type (a frameshift mutation is produced either by insertion or deletion of one or more new bases; Splicing: SNV annotated to 2 bp regions on both sides of the intronic region; Stop-gain: SNV in which the codon where the base is located is changed into a stop codon due to the substitution of the base of nonsynonymous mutation; Nonsynonymous: Nonsynonymous SNV). Exon: Exon where the mutation is located; Position: Chromosome where the mutation is located and its position; hgvs.c: Nucleotide change caused by the mutation; Genotype: Genotype of the locus; het: Heterozygous, hom: Homozygous; ACMG level: ACMG grading result; SNV: Single nucleotide variant; ACMG: American College of Medical Genetics and Genomics.

**Table 5:** Correlation analysis results of mutation loci of candidate susceptibility genes of severe periodontitis

**Gene**	**Candidate variants**	**Case, n** **(AF %)**	**Control, n** **(AF %)**	**dbSNP 155, n (AF %)**	***P* value^1^**	***P* value^2^**
*LFNG*	rs34637446 (GATA>deletion/insertion)	15 (53.33)	21 (52.38)	34 (50.00)	0.936	0.761
*LENG8*	rs10407651+TAG+rs1035451 (ATAGG>ATAGA) (ATAGG>GTAGA)	15 (26.67) 15 (13.33)	21 (35.71) 21 (9.52)	– –	0.417 0.899	– –
*NPHS1*	rs114615449 (G>C)	15 (10.00)	21 (2.38)	112 (1.80)	0.104	0.038^*^
	rs28939695 (G>A)	15 (10.00)	21 (4.85)	112 (2.70)	0.389	0.042^*^
*HFE*	rs1799945 (C>G)	15 (6.78)	21 (4.85)	–	0.728	–
*ILDR1*	rs142746163 (C>T)	15 (6.78)	21 (2.38)	112 (0.00)	0.360	0.014^*^
*DMXL2*	rs3078066 (AAAAAAAAAAAAAAAAAAAA>A >deletion/insertion)	15 (6.78)	–	54 (0.00)	–	0.046^*^

Asterisks indicate statistical significance. *n* refers to the number of participants; *P* value^1^ refers to comparisons between case and control groups; *P* value^2^ refers to comparisons between case and dbSNP 155 control groups. AF: Allele frequency; SNP: Single nucleotide polymorphism.

### Sanger sequencing verification

**Figure 3.**
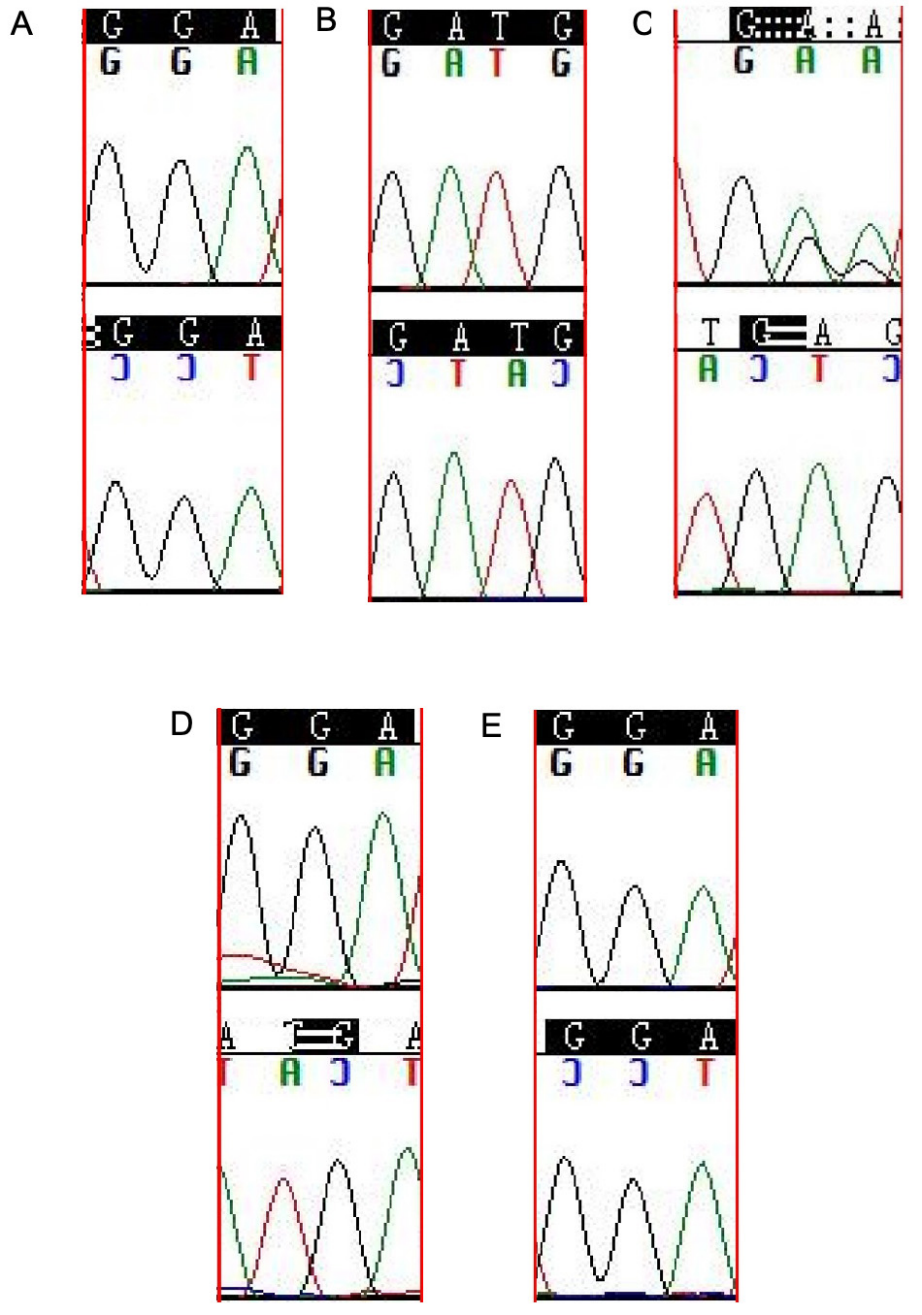
**Sanger sequencing chromatogram of rs34637446 mutation type of** *LFNG.* (A) *LFNG* heterozygous mutant (GATG insertion); (B) *LFNG* homozygous mutant (GATA insertion); (C) *LFNG* heterozygous mutant (GATG deletion); (D) *LFNG* homozygous mutant (GATA deletion); (E) *LFNG* wild-type.

**Figure 4.**
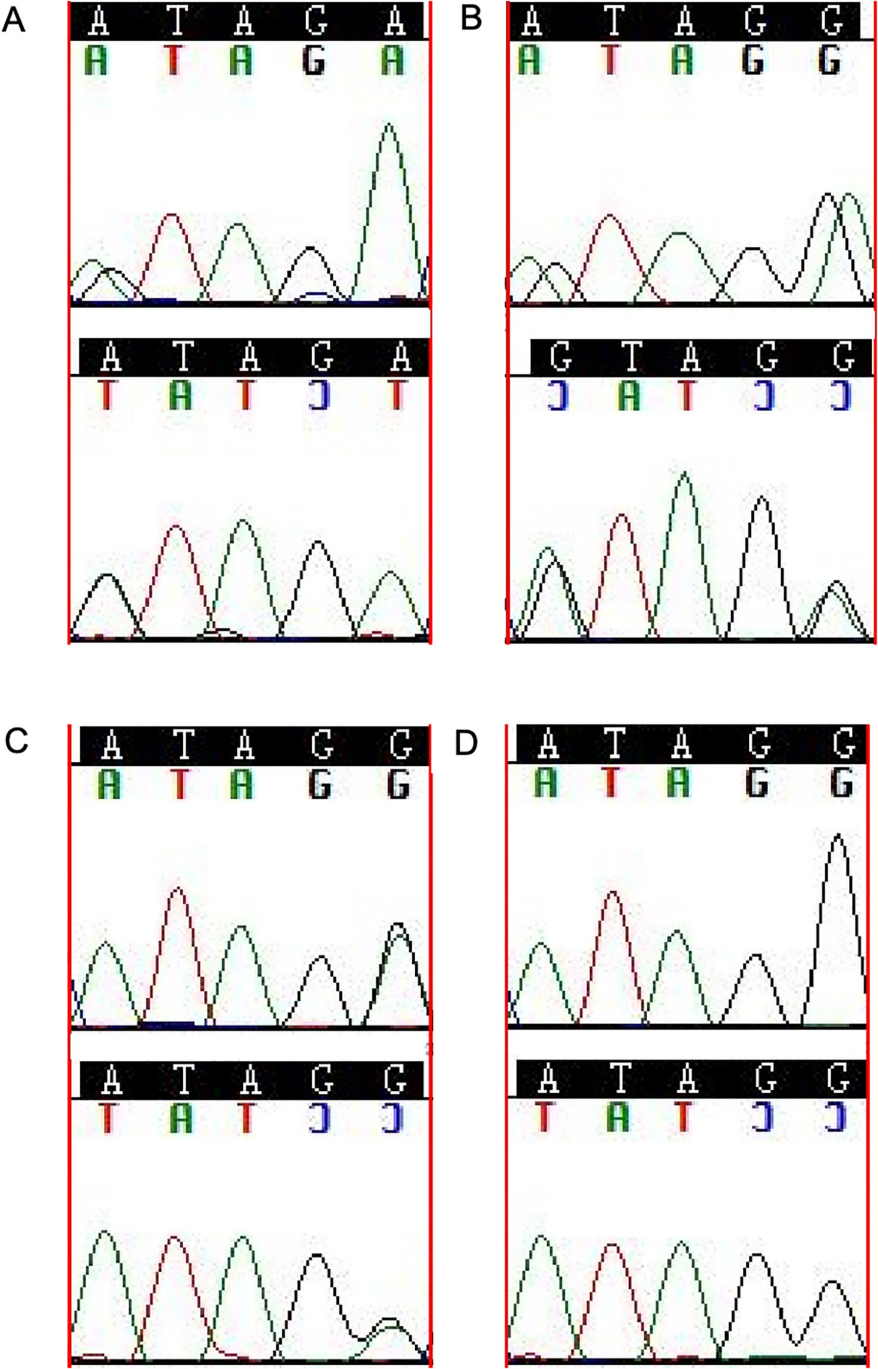
**Sanger sequencing chromatogram of rs10407651+TAG+ rs1035451 mutation type of ***LENG8.* (A) *LENG8* heterozygous mutant (ATAGA/GTAGA); (B) *LENG8* heterozygous mutant (ATAGG/GTAGA); (C) *LENG8* heterozygous mutant (ATAGG/ATAGA); (D) *LENG8* wild-type (ATAGG/ATAGG).

We found that all SNV/SNP loci were consistent with the WES results. However, the WES and Sanger results differed in InDel loci. According to the dbSNP 155 database, exon2: c.163_166dupGATG in *LFNG* was a major variation type of nucleotide polymorphism locus rs34637446 which mainly included exon2: c.163_166dupGATG and exon2: c.163_166delGATG. Our Sanger verification showed that 12 of 15 samples had mutations (including 6 cases of GATG heterozygous deletion, 2 cases of GATG heterozygous insertion, 1 case of GATG homozygous deletion, and 3 cases of GATG homozygous insertion) (Figure 3). WES of *LENG8* found that the InDel locus exon2: c.1-59_1-55delinsGTAGA was a mutation locus consisting of rs10407651+TAG+rs1035451, with a short nucleotide sequence ATAGG>GTAGG/ATAGA/GTAGA and located in the splicing region. ATAGG> mutations were found in 10 out of 15 samples, including 8 cases of ATAGA heterozygous mutation and 4 cases of GTAGA compound heterozygous mutation; of these, 2 cases had both ATAGA and GTAGA heterozygous mutations present (Figure 4). DMXL2 c.88-3dup was an InDel locus, and the nucleotide polymorphism locus rs3078066 mainly manifested as single or multiple A insertions or deletions, primarily in the form of wild type “AAAAAAAAAAAAAAAAAAAA” (*n* ═ 7810, Group (Global) 100%). Our WES results could not be verified by first-generation Sanger sequencing due to the presence of long nucleotide sequence repeats.

### Correlation analysis

The allele frequencies at the mutation loci of *LFNG, LENG8, NPHS1, HFE, ILDR1,* and *DMXL2* genes were analyzed in the 15 patients with severe periodontitis. The correlations between the allele frequencies and those reported in the dbSNP 155 database (East Asian race), and in our healthy control group were calculated. We found that the allele loci of *NPHS1*(rs114615449), *ILDR1*(rs142746163), and *DMXL2*(rs3078066) showed significant differences between the severe periodontitis patients and dbSNP 155 (East Asian race) (*P* < 0.05) (Table 5).

## Discussion

In this study, we aimed to identify susceptibility genes for stage III and IV periodontitis using on WES followed by confirmatory Sanger sequencing. We obtained saliva samples from 15 patients with stage III or IV periodontitis, from which DNA was extracted and analyzed for quality. The total amount of DNA extracted reached the standard of WES (concentration ≥3 ng/µL, total amount ≥200 ng), and the extracted DNA was free of contamination (grade 1). These results indicate that the quantity and quality of genomic DNA extracted from saliva can be considered equivalent to blood for WES, suggesting that the application of saliva DNA extraction is a convenient, easy, and reliable approach that provides a non-invasive alternative source of genomic DNA for genetic analysis.

Sanger sequencing verification was performed on the mutation loci of candidate susceptibility genes associated with severe periodontitis screened by WES. It was found that all SNV/SNP loci were consistent with the WES results, but the WES result of the InDel locus differed from the Sanger sequencing results, suggesting that the accuracy of WES for small-fragment nucleotides is lower than for single nucleotides, and sequencing depth and coverage may have an impact on the accuracy of small-fragment nucleotide variations. In this study, false negative results (*LFNG* and *LENG8*) were found using WES for small-fragment nucleotide variations, although they may still indicate the presence of InDel or small-fragment nucleotide variants but need to be verified by Sanger sequencing.

According to the screening of low-frequency and harmful mutations, the pathogenicity predictions based on the ACMG criteria, and shared mutation analysis results, InDel mutations (GATA>deletion/insertion) at *LFNG* rs34637446 were found in 12 out of 15 patients with severe periodontitis. Using dbSNP and our control group, we confirmed that the frequency of the rs34637446 mutation is high in Han Chinese individuals. Based on our correlation analysis, we rejected *LFNG* as a candidate gene. Twelve of the 15 cases showed *LENG8* mutation loci consisting of rs10407651+TAG+rs1035451 with a short nucleotide sequence ATAGG>GTAGG/ATAGA/GTAGA. There were 8 cases of ATAGG>ATAGA mutation and 4 cases of ATAGG>GTAGA compound heterozygous mutation. However, our correlation analysis dismissed *LENG8* as a candidate gene. Two cases had an *HFE* mutation at exon2: c.187C>G. The *HFE*-encoding protein is a membrane protein that regulates iron uptake by regulating the interaction between the transferrin receptor and transferrin. *HFE* gene deficiency leads to hereditary hemochromatosis, a recessive iron storage disorder [22, 23]. The correlation analyses with the control group also rejected *HFE* as a candidate gene.

Shared mutations of *ILDR1* were found at exon6: c.772C>T in two cases. *ILDR1* encodes an immunoglobulin-like domain-containing protein that can function as a multimeric receptor on the cell surface. It has been mostly reported that *ILDR1* mutations are associated with hearing loss or deafness [24]. The shared mutations of *NPHS1* in 3 cases were found at exon 14: c.1802G>C and exon11: c.1339G>A. *NPHS1* encodes a member of the immunoglobulin family of cell adhesion molecules that functions in the glomerular filtration barrier in the kidney, and its mutations are associated with nephrotic syndrome [25, 26]. NPHS1 is primarily expressed in the kidney, lymph nodes, and pancreas. In this study, our correlation analysis with dbSNP showed that *ILDR1* and *NPHS1* were associated with severe periodontitis (*P* < 0.05). However, correlation analysis with the control group rejected *ILDR1* and *NPHS1* as candidate genes. Moreover, the gene function of *ILDR1* and *NPHS1* was predicted based on the ARCHS4 (https://maayanlab.cloud/archs4), and we found that neither *ILDR1* nor *NPHS1* result in the human “periodontitis” phenotype.

Current understanding suggests that the tissue injury observed in periodontitis mainly results from an abnormal host immune response to microbial infection [27, 28]. Immune cells play an important role in regulating immune responses and maintaining immune stability. Tacrolimus, a calcineurin inhibitor, has been demonstrated to protect against the inflammation-induced tissue and bone loss associated with periodontitis in experimental rats and oral diseases through a mechanism involving IL-1β, TNF-α, and IL-6 [29, 30]. In this study, we identified a *DMXL2* splicing mutation at rs3078066 in two cases. These mutations describe a process where introns are removed following DNA transcription into RNA. During the splicing process, abnormalities appear when a mutation occurs at a locus, altering the protein function. Splicing mutations mainly result in two outcomes. First, an mRNA frameshift occurs, which, similar to the frameshift mutation, often leads to nonsense-mediated mRNA decay or produces truncated proteins, resulting in pathological outcomes. Second, no mRNA frameshift occurs, altering the number of amino acids in the mutant protein. The ultimate effect on protein function depends on the position of the insertion or deletion. Specifically, pathology is likely to occur if the amino acids are located in the important functional domains of the protein. *DMXL2* encodes a protein with 12 WD domains. Proteins with WD domains are involved in many functions including participation in signal transduction pathways. *DMXL2* is involved in regulating the Notch signaling pathway (CITE) which controls IL-1β and TNF-α activities [31] and inhibits other inflammatory cytokines [32]. Notch receptors are closely related to regulatory T cells [33]. Regulatory T cells are a unique subpopulation of CD4^+^ T cells with immunosuppressive activity that prevents inflammatory injury by inhibiting autoimmunity and regulating the immune response [34, 35]. According to our gene function prediction analysis based on ARCHS4, *DMXL2* may cause the human “periodontitis” phenotype. Therefore, we speculate that the *DMXL2* gene mutation at the exon level alters the host immune defense response in some patients with severe periodontitis, ultimately causing severe periodontitis. Given the possible association between *DMXL2* and severe periodontitis genes, large-sample verification is needed.

Based on the number of cases with shared mutations and correlation analysis results, we found that the *DMXL2* gene might be associated with stage III and IV periodontitis. We speculate that mutations in this gene at the exon level might alter the host immune defense response, and cause severe periodontitis and its progression by dysregulating the Notch signaling pathway. Consequently, we suggest the *DMXL2* gene as a possible new candidate susceptibility gene for severe periodontitis. The results of this study remain to be verified by larger studies to determine the pathogenic gene mutation or polymorphism locus of severe periodontitis. Our study shows a possible effect of a *DMXL2* gene mutation on the risk of stage III and IV periodontitis among Han Chinese individuals, which now needs confirmation in other ethnic groups to determine its generalizability and further experimentation to clarify all involved mechanisms.

## Conclusion

In the last few decades, many studies have been performed to identify periodontitis susceptibility genes, which may be useful to find even more potential risk and therapeutic targets for periodontitis. In the present study, we first identified heterozygous mutation loci in the *DMXL2* gene in 2 of 15 cases with stage III and IV periodontitis by WES, which shows a possible effect of this gene mutation on the risk of severe periodontitis among Han Chinese individuals. Our results indicate that *DMXL2* may be a susceptibility gene for the development of severe periodontitis, but further large-sample studies are needed to confirm the results. Furthermore, we proved that extraction of DNA from the saliva provides an alternative to the peripheral blood for WES, which is beneficial for clinical research and application of WES in the population with a genetic disease. Our study provides novel insight and supports the feasibility of conducting large-sample studies on susceptibility genes, gene polymorphisms, and gene mutations in periodontitis.
